# Correction: Sławek et al. Case Series and Literature Review on Botulinum Toxin Efficacy in Axial Extensor Truncal Dystonia. *Toxins* 2025, *17*, 375

**DOI:** 10.3390/toxins17090435

**Published:** 2025-09-01

**Authors:** Jarosław Sławek, Iga Alicja Łobińska, Michał Schinwelski, Joanna Kopcewicz-Wiśniewska, Anna Castagna

**Affiliations:** 1 Division of Neurological-Psychiatric Nursing, Faculty of Health Sciences, Medical University of Gdańsk, 80-210 Gdańsk, Poland; 2 Department of Neurology & Stroke, St. Adalbert Hospital, Copernicus Ltd., 80-462 Gdańsk, Poland; 3 Medical Faculty, Medical University of Gdańsk, 80-210 Gdańsk, Poland; 4 Neurocentrum-Miwomed, Neurological Clinic, 80-207 Gdańsk, Poland; 5 IRCCS Fondazione Don Carlo Gnocchi, Onlus, 20162 Milan, Italy

The authors wish to make the following corrections to this paper [1]. In the original publication, video segments were not properly cited in the Supplementary Materials section, and the videos were left out due to a mistake in the processing of the manuscript. The authors state that the scientific conclusions are unaffected. The correction was approved by the Academic Editor. The original publication has also been updated.

## Error in Content

### 3.1. Case No. 1

“Video Segment 1” should be corrected as “Video No. 1 Segment 1 in Supplementary Materials”.

“Video Segment 2” should be corrected as “Video No. 1 Segment 2 in Supplementary Materials”.

### 3.2. Case No. 2

“Video Segment 1” should be corrected as “Video No. 2 Segment 1 in Supplementary Materials”.

“Video Segment 2” should be corrected as “Video No. 2 Segment 2 in Supplementary Materials”.

“Video Segment 3” should be corrected as “Video No. 2 Segment 3 in Supplementary Materials”.

### 3.3. Case No. 3

“Video Segment 1” should be corrected as “Video No. 3 Segment 1 in Supplementary Materials”.

“Video Segment 2” should be corrected as “Video No. 3 Segment 2 in Supplementary Materials”.

### 3.4. Case No. 4

“Video Segments 1 and 2” should be corrected as “Video No. 4 Segments 1 and 2 in Supplementary Materials”.

“Video Segment 3” should be corrected as “Video No. 4 Segment 3 in Supplementary Materials”.

## Error in Back Matter

The Supplementary Materials needed to be added as follows.

**Supplementary Materials:** The videos can be downloaded at: https://zenodo.org/records/16932212?token=eyJhbGciOiJIUzUxMiJ9.eyJpZCI6ImE4YWE2ZjAwLTA3ZGYtNDQzYi1iZjIwLTAyNDlkMTU2OTU5MCIsImRhdGEiOnt9LCJyYW5kb20iOiIxN2Y1NTI1ODYyNGYyNzljYTc1MzRhMmE3OTJiMDA5YyJ9.Zui0ZSD6eVhbDIbcNAgLiEQL3eQq-Iq92a7OjvLmjYqu6UFK0hPyJ_QjPBDtpx-l9ukz4mJo4wczUnqghdPkDA (accessed on 10 April 2025).
